# Findings of Reduced Head Circumference with COVID-19 Infection in the Third Trimester: A Retrospective Cohort Study

**DOI:** 10.3390/biomedicines13040832

**Published:** 2025-03-31

**Authors:** Kristen Lee Moriarty, Kelsey Manfredi, Pascale Carrel, Emma Kryzanski, David A. Schwartz, Lucas Godoy, Chia-Ling Kuo, Andrea Shields

**Affiliations:** 1 Department of Obstetrics & Gynecology, Division of Maternal-Fetal Medicine, UConn Health, Farmington, CT 06030, USA; manfredi@uchc.edu (K.M.); ashields@uchc.edu (A.S.); 2 School of Medicine, The University of Connecticut, Storrs, CT 06030, USA; carrel@uchc.edu (P.C.); kryzanski@uchc.edu (E.K.); 3 Perinatal Pathology Consulting, Atlanta, GA 30342, USA; davidalanschwartz@gmail.com; 4 The Cato T. Laurencin Institute for Translation in Regenerative Engineering, UConn Health, Farmington, CT 06030, USA; ldacunha@ucsc.edu (L.G.); kuo@uchc.edu (C.-L.K.)

**Keywords:** fetal growth restriction, COVID-19, head circumference, fetal growth, small for gestational age, SARS-CoV-2, pregnancy, placental dysfunction, fetal brain growth, prenatal exposure to COVID-19, maternal–fetal health, maternal infection, neurodevelopmental impact, impact of viral infections on pregnancy

## Abstract

**Background**: COVID-19 is linked to multiple adverse pregnancy outcomes but with inconsistent evidence associating the disease with fetal growth restriction (FGR) and small for gestational age (SGA). There are limited data on the impact of COVID-19 on neonatal growth measurements, specifically microcephaly without SGA or low birth weight. We hypothesize that COVID-19 is associated with smaller neonatal head measurements without increasing the risk of small for gestational age. This relationship may be related to the timing of COVID-19 exposure in pregnancy. **Methods**: An Institutional Review Board (IRB) approved retrospective cohort study enrolled 140 COVID-19-infected and 136 COVID-19-uninfected patients. Inclusion criteria: (a) singleton birth between 28 April 2020 and 31 December 2022; and (b) maternal COVID-19 infection diagnosed via polymerase chain reaction (PCR). Exclusion criteria: Less than 12 years of maternal age, major fetal anomalies, and fetal loss < 15 weeks. The outcomes were a comparison of newborn growth measurements (length, weight, and head circumference (HC) at birth), Ponderal Index (PI), and development of SGA between SARS-CoV-2-infected and uninfected patients. Maternal and neonatal characteristics were descriptively summarized, and multivariate analyses and linear regression models were performed. Baseline maternal demographics did not differ amongst cohorts. **Results**: Compared to the uninfected cohort, COVID-19 diagnosed in the third trimester was associated with a lower neonatal HC compared to newborns of uninfected patients (β = −0.38 [0.38 SD lower], 95% CI −0.65 to −0.10, *p* = 0.024). There was no significant difference among cohorts in birth length, weight, or diagnosis of small for gestational age. **Conclusions**: We found that COVID-19 infection in the third trimester was associated with a lower neonatal head circumference without associated SGA. The cause underlying this association is unknown. Further research to determine the risk of neurotropic fetal infection by SARS-CoV-2, like ZIKA’s effect on the fetal immune system leading to microcephaly, is urgently needed.

## 1. Introduction

Coronavirus disease 2019 (COVID-19) has been linked to multiple adverse pregnancy outcomes (APOs) for both the mother and fetus, including preterm delivery, miscarriage, stillbirth, pre-eclampsia-like syndrome, and abnormal fetal growth and development [1,2,3,4,5,6]. These APOs result in a greater need for neonatal intensive care unit (NICU) admissions, comorbidities of asphyxia-related complications, hyperbilirubinemia, and increased perinatal mortality. For example, a systematic review reported increased odds for the development of women infected with COVID-19 for pre-eclampsia (odds ratio (OR) 1.33), preterm birth (OR 1.82), and stillbirth (OR 2.11) [7]. Moreover, The INTERCOVID Multinational Cohort Study found that pregnant women with COVID-19 are twenty times more likely to die compared to unaffected pregnant women [8].

Recent data demonstrate that patients infected with the Severe Acute Respiratory Syndrome Coronavirus-2 (SARS-CoV-2) can develop a placental infection, termed SARS-CoV-2 placentitis, that causes an average of 77% placental destruction and consequent placental insufficiency among stillbirths and early neonatal deaths [3,9]. However, the literature is inconsistent on the impact of SARS-CoV-2 infection during pregnancy and the growth of the fetus [2,4,5,6,10,11,12,13]. Some viral agents have been reported to cause fetal growth and development abnormalities. For example, Cytomegalovirus is the most common cause of fetal growth restriction (FGR) [14]. Still, other viruses, including Zika virus (ZKV) [15], rubella virus [16], and varicella-zoster virus [17], are associated with abnormal fetal growth and development. In particular, the Zika virus has been associated with the diagnosis of disproportionate microcephaly without associated small for gestational age (SGA) [15,18,19,20,21]. Fetal head circumference (HC) enlarges by approximately 1 mm/day between 26 weeks of gestation and 32 weeks and about 0.7 mm/day between 32 and 40 weeks [22]. Given the importance of fetal neurodevelopment, early recognition and knowledge of specific viruses that cause neurotropic fetal infections are paramount. Because the SARS-CoV-2 pandemic did not start until 2020, there are limited data on the effects of COVID-19 during pregnancy and newborn growth measurements, specifically head measurements of the newborn in the absence of SGA. Yet, research has shown that COVID-19 can cause neurotrophic derangements.

SARS-CoV-2 virus enters the central and peripheral nervous system and infects pericytes and astrocytes, compromising the blood–brain barrier and spreading to vital brain structures [23]. Studies have found increased serological biomarkers associated with CNS damage in patients with COVID-19, such as increased plasma glial fibrillary acidic protein and neurofilament light chain [24]. Brain imaging performed at the time of death from patients infected with COVID-19 showed white matter changes, posterior reversible encephalopathy syndrome, and more [25]. Moreover, neurological damage has been documented from extensive COVID-19 infections in the fetus [26,27,28,29]. In 2023, a case report noted massive cerebral hemorrhage in a fetus with severe maternal COVID-19 infection with post-mortem histologic neural analysis showed massive intervillous deposition of fibrin and inflammatory infiltration with hotspots of necrotic deciduitis [26]. Given the documented neurological insult potential for SARS-CoV-2, it has become apparent the need to further understand changes in growth parameters in pregnancies complicated by COVID-19.

The primary objective was to investigate the relationship between trimester-specific SARS-CoV-2 infection and newborn growth metrics. We hypothesize that COVID-19 may result in a disproportion between neonatal head measurements and weight without increasing the risk of SGA and may be associated with the timing of COVID-19 exposure in pregnancy.

## 2. Materials and Methods

**Figure 1:**
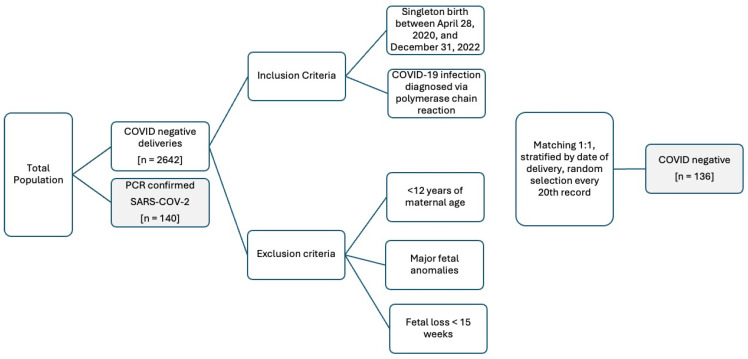
Demographics for selection of study population for the retrospective cohort study for 140 COVID-19-infected and 136 COVID-19-uninfected patients.

A retrospective cohort study was performed at an academic tertiary hospital in the Northeast region of the United States. Our study received approval from our hospital’s institutional review board (IRB #23X-086-2) and met all ethical standards by the Declaration of Helsinki. Medical records were obtained from the electronic medical record system (EMR). The inclusion criteria included the following: (a) singleton pregnancy; (b) birth occurring at our institution between 28 April 2020 and 31 December 2022; and (c) maternal COVID-19 infection diagnosed via PCR. Exclusion criteria included <12 years of maternal age, major fetal anomalies, and fetal loss <15 weeks. All patients in the study had a first or second-trimester ultrasound used to establish or confirm their estimated due date. A total of 140 COVID-19-positive patients were delivered to the home institution within the time frame and met all inclusion and exclusion criteria. A total of 2642 pregnant patients were delivered to the home institution between February 2020 and August 2022. In total, 136 COVID-negative patients were matched in a 1:1 ratio from the institution’s 2642 deliveries during the time frame by stratifying by date and selecting every twentieth patient for inclusion in the analysis. Thus, our final cohorts were split between 136 patients in the COVID-negative group and 140 in the COVID-19-infected group as shown in Figure 1.

Study data were collected using Research Electronic Data Capture (REDCap), a secure, web-based application designed to support data capture for research studies [30,31]. A core outcome set was not used in the initial study design. Baseline maternal demographics included maternal age at delivery, gravidity, and parity, recreational drug use, pre-pregnancy BMI (body mass index), pre-existing maternal medical conditions; gestational age, weight, and percentile at anatomy scan (to account for constitutional differences in fetal growth during pregnancy); gestational age at delivery, mode of delivery, indication(s) for delivery, COVID-19 vaccination status, gestational age and symptoms at the time of COVID 19 diagnosis, hospitalization at the time of COVID-19 diagnosis, and receipt of treatment for COVID-19. Outcomes data collected included growth measurements (length, weight, and HC at birth), gender of the neonate, Apgar scores at delivery, and NICU admission. Outcomes consisted of collecting and comparing newborn growth measurements (length, weight, and head circumference (HC) at birth), PI, and SGA between COVID-19-exposed and non-exposed pregnancies. The definition of SGA is a newborn whose birthweight <10th percentile for gestational age and gender as defined by the Society of Maternal Fetal Medicine [32]. The growth percentile at birth was assigned based on the 2013 Fenton Preterm Growth Chart [33], which considers weight, length, and HC adjusted for gender and gestational age at birth. The Ponderal index (PI), a calculated metric of body proportionality that factors the relationships between weight and length, is determined for every neonate to distinguish between symmetric versus asymmetric growth restriction [34,35]. The PI formula is [birth weight (in g) × 100] ÷ [birth length (in cm)^3^]. A PI less than the 10th percentile (adjusted for gestational age) is diagnostic of asymmetric growth restriction, and a PI of less than the 3rd percentile indicates severe fetal wasting. Other neonatal outcomes collected included APGAR scores and admission to the NICU. Maternal and neonatal characteristics were descriptively summarized, and multivariate analyses and linear regression models were performed.

Maternal and neonatal characteristics and infant growth outcomes were descriptively summarized overall. Pregnant women infected with COVID-19 were grouped by time of diagnosis and compared to COVID-19 uninfected women. Kruskal–Wallis test was used to compare the infected and uninfected cohorts for continuous and ordinal variables, and the Chi-square test or Fisher’s exact test, used for smaller numbers where appropriate, was used to analyze categorical variables. Significant associations with the cohorts (*p* < 0.05) were jointly examined in a multiple linear regression model. The adjusted mean differences comparing the COVID-19-infected cohorts to the uninfected cohort were reported, including the 95% confidence intervals and *p*-values; *p*-values less than 5% were considered statistically significant. All the statistical tests were two-sided, and the statistical analyses were performed in R version 4.2.2.

## 3. Results

The maternal baseline demographic data for the entire study population, including both COVID-19-infected (n = 140) and COVID-19-uninfected (n = 136) patients, is represented in Table 1. Twenty-seven percent (n = 74) of the population self-identified as Hispanic, and 72% (n = 199) self-identified as non-Hispanic. Forty-four percent (n = 122) self-identified as White, 22% (n = 60) as African American, and 23% (n = 64) as “other”. The “other” category encompasses Native Hawaiian or Pacific Islander, unknown race, or patient-reported “other”, which may include multiple racial identities. The mean pre-pregnancy BMI was 29.01 ± SD 7.52, and the mean maternal age at delivery was 30.41 years ± SD 5.26. Twenty-five percent (n = 49) of patients had a hypertensive disorder of pregnancy; 6% (n = 17) had chronic hypertension, 10% (n = 27) had gestational hypertension, 4% (n = 10) had pre-eclampsia with severe features, 2% (n = 5) had pre-eclampsia without severe features, and 3% (n = 8) had superimposed pre-eclampsia with severe features. Thirteen percent (n = 22) of patients had diabetes; one patient had type 1 diabetes, 3% (n = 8) had type 2 diabetes, 5% (n = 14) had GDMA1 (diet-controlled gestational diabetes), and 5% (n = 13) had GDMA2 (gestational diabetes treated with medication). Very few patients reported drug use of any kind, with the highest percentage (9%, n = 26) reporting cannabis use. Three percent of patients (n = 7) had a history of prior FGR. Sixty-six percent (n = 181) of infants were born via vaginal delivery, 33% (n = 90) via cesarean delivery, and 2% (n = 5) via vaginal birth after cesarean delivery. There was no significant difference in the mode of delivery (*p* = 0.413) or gestational age at delivery (*p* = 0.199) among maternal COVID-19-infected and -uninfected groups.

Table 1 further stratifies maternal demographic data by trimester-specific timing of infection with COVID-19: COVID-19 diagnosis before 13 weeks (first trimester), between 13 and 28 weeks (second trimester), and between 28 and 42 weeks (third trimester). There was a trend toward a history of pregnancy-induced hypertension for patients with a diagnosis of COVID-19 in the third trimester (*p* = 0.074). Otherwise, maternal baseline demographics were similar between cohorts.

Table 2 shows newborn outcome data, including infants born to COVID-19-infected (n = 140) and COVID-19-uninfected (n = 136) patients. All infants were singleton gestations. Infants were evenly distributed by gender, with 49% female (n = 135) and 51% male (n = 141). Thirteen percent (n = 37) of infants were admitted to the NICU. The mean birth percentile growth measurements reported in the Z score are summarized for the total population with weight −0.16 ± SD 0.88, HC −0.17 ± SD 0.98, length 0.19 ± SD 1.08. The median PI was 2.54 (range 1.62 to 3.37). Note that a normal PI for a newborn varies between 2.2 and 3.0, depending on gestational age. Eight percent (n = 21) had a diagnosis of SGA, and 13% (n = 35) had a diagnosis of FGR. Table 2 also stratifies infant demographic data by timing of maternal COVID-19 diagnosis: diagnosis before 13 weeks (first trimester), between 13 and 28 weeks (second trimester), and between 28 and 42 weeks (third trimester). The COVID-19 groups were significantly associated with lower infant HC percentile (in z scores) in the third trimester (*p* = 0.010). Newborns from patients affected with COVID-19 between 28 and 42 weeks had a significantly lower mean HC vs. newborns from COVID-19 uninfected patients (β = −0.49 ± 0.92, *p* = 0.010). There was no statistical significance for mean PI (*p* = 0.417), birth weight (*p* = 0.431), or birth length (*p* = 0.577). Neither a diagnosis of SGA (*p* = 0.878) nor FGR (*p* = 0.496) were statistically significant between groups. There was a trend toward a higher prevalence of female compared to male infants affected with COVID-19 in the third trimester compared to the first and second, although not statistically significant (*p* = 0.092). Additionally, the gestational age at anatomy scan differed between the trimester of COVID-19 diagnosis, with infants in the third trimester with maternal infection with COVID-19 having slightly increased gestational age at anatomy scans compared to the other cohorts (*p* = 0.043).

Significant associations between the COVID groups and growth outcomes were further examined in multivariate analyses, as shown in Table 3. Linear regression models were fitted for the growth outcomes comparing COVID moms by diagnosis time to non-COVID moms, adjusting for covariates selected because of significant associations with COVID groups at a 10% significance level. In the multivariate analysis, adjusting for gestational age at diagnosis, gestational age at anatomy scan was no longer statistically significant (*p* = 0.3). However, infant head HC remained significantly lower with COVID-19 patients diagnosed in the third trimester vs. non-infected patients (β = −0.38 [0.38 SD lower], 95% CI −0.65 to −0.10, *p* = 0.024).

## 4. Discussion

Our study found that COVID-19 diagnosed in the third trimester had a significant association with newborn growth measurements, specifically the development of small HC, compared to patients unaffected by COVID-19. Although our study did not show an abnormal PI in COVID-19-affected patients, the calculation of the PI only factors neonatal length and weight and not head circumference, which limits its application to determine asymmetric growth issues in pregnancies affected by COVID-19. Our study did not show an association of maternal COVID-19 with the development of SGA.

Our study has some limitations as follows: (1) We did not examine specific SARS-CoV-2 variants in the cases presented, and different virus variants may have varying impacts on maternal and fetal health. (2) Based on the retrospective data collection, we do not have data on parental head circumference, which can strongly influence neonatal head circumference. It has been reported that 50% of head size variation in newborns is familial; hence, adjusting for parental head size is essential [36]. (3) We did not examine the long-term neurodevelopmental outcomes of the newborns. Understanding the neurotropic impact of the SARS-CoV-2 virus beyond a newborn head circumference is critical. (4) We also did not examine placental pathology as it was not reported in all cases; COVID-19 has resulted in placental tissue destruction and insufficiency, an essential cause of growth restriction [37]. Understanding the effects of COVID-19 on the placental tissue—especially tissue destruction and insufficiency—could provide insights into the mechanisms behind growth restriction and further our understanding of the maternal–fetal impact of the virus. (5) Although our baseline maternal and infant baseline demographic data showed minimal differences between cohorts, differences between groups may be appreciated with larger sample sizes. The gestational age at the anatomy scan differed between cohorts. However, there was no difference between the estimated fetal weight or the percentile at the anatomy scan. In our practice, almost all patients have an established due by the first-trimester ultrasound.

Studies have inconsistently shown a link between COVID-19 and the development of FGR or SGA. However, little is known about COVID-19 and the potential for altered fetal head development and microcephaly. Steiner et al. demonstrated that infants born to women requiring hospitalization for COVID-19 had lower birth weight (*p* < 0.01), shorter birth length (*p* = 0.02), and smaller HC (*p* = 0.03) compared to infants born to COVID-19-infected women who did not require hospitalization [38]. Additionally, The INTERCOVID Multinational Cohort Study demonstrated that patients diagnosed with COVID-19 were more likely to deliver infants with lower birth weight (*p* < 0.001), length (*p* < 0.01), and HC (*p* < 0.01) than uninfected patients [8]. Farrell et al. found that the trimester in which pregnant patients contracted COVID-19 had no significant effects on the birth weight, customized birth weight percentiles, or prevalence of SGA among newborns [1]. However, infants born to patients having symptomatic COVID-19 infection had significantly lower mean birth weight and median birth weight percentiles compared to infants born to infected women who were asymptomatic. They also found a higher prevalence of SGA among patients infected with COVID-19 than in previous studies, with one possible explanation being vertical transmission of COVID-19 from mother to fetus, irrespective of gestational age at diagnosis. A longitudinal cohort study by Ockene et al. found that fetuses exposed to COVID-19 in utero had a lower BMI after delivery than unexposed fetuses, even after adjusting for gestational age and other potential confounders. Infants born to COVID-19-infected patients also had a more rapid increase in BMI in their first 12 months of life, with the steepest rate of rise in infants born to patients with the most severe infection. Differences in body length between infants exposed and unexposed to COVID-19 were not statistically significant [39].

Fetal head growth in the third trimester contributes substantially to the final head circumference at birth. Disruption of fetal HC growth has been associated with neurodevelopmental abnormalities, as has been seen with other viral infections [15,18,19,20]. Specifically, the Zika virus, a flavivirus, has received great attention due to its association with microcephaly [15,18,19,20]. Zika virus cases were first documented in 2015 when providers in Brazil noted an increase in fetuses born with microcephaly. A systemic review by Antoniou et al. analyzed 15 articles published from 2018 to 2021 and found the incidence rate of ZKV more common than expected at 15% of pregnancies [19]. The definition of microcephaly is challenging and not formally explicit. Most studies agree that the diagnosis of microcephaly can be made if the mean head circumference is two standard deviations below the mean for gestational age. Microcephaly can arise from viral infection, insufficient neural stem cell development, and eventual neuronal cell death [18,37].

## 5. Conclusions

Our study found that COVID-19 in pregnancy was associated with smaller HC if diagnosed in the third trimester independent of the diagnosis of SGA or a ponderal index <10%. Since fetal head circumference dramatically increases in the third trimester, our study found that a COVID-19 diagnosis in the third trimester is associated with smaller head circumference. This information highlights the need for further research to investigate the relationship between COVID-19 during pregnancy and its neurotropic impact on fetal head growth and long-term neurodevelopmental outcomes, particularly with third-trimester exposure. Continued research in these areas will contribute to a more comprehensive understanding of how maternal COVID-19 infection affects fetal and neonatal neurodevelopment and growth, enabling better care and management for pregnant individuals and newborns. Future studies should aim to expand the data collection to include parental head circumference, gathering data on neurodevelopmental outcomes between groups, and examining the influence of specific viral variants on fetal growth and other outcomes.

## Figures and Tables

**Table 1:** Maternal Outcome Data for Total Population and Stratification by Trimester in COVID-19 Exposed Pregnancies.

			COVID Diagnosis			
	Population (N = 276) ^1^	No COVID (N = 136)	<13 w (N = 11)	13 to 28 w (N = 54)	28 to 42 w (N = 75)	Value ^2^
Gravity (N = 276)						0.440
Mean ± standard deviation	2.63 ± 1.75	2.52 ± 1.68	2.64 ± 1.50	2.91 ± 1.91	2.64 ± 1.81	
Median; Range	2.00; 0.00 to 11.00	2.00; 0.00 to 8.00	3.00; 1.00 to 6.00	2.00; 1.00 to 11.00	2.00; 1.00 to 9.00	
Parity (N = 276)						
0	117 (42%)	61 (45%)	5 (45%)	18 (33%)	33 (44%)	
1	89 (32%)	40 (29%)	5 (45%)	21 (39%)	23 (31%)	
2	48 (17%)	26 (19%)	1 (9%)	10 (19%)	11 (15%)	
3	18 (7%)	6 (4%)	0 (0%)	4 (7%)	8 (11%)	
4	3 (1%)	2 (1%)	0 (0%)	1 (2%)	0 (0%)	
5	1 (0%)	1 (1%)	0 (0%)	0 (0%)	0 (0%)	
Ethnicity (N = 276)						0.344
Hispanic	74 (27%)	32 (24%)	3 (27%)	16 (30%)	23 (31%)	
None or Unknown	3 (1%)	1 (1%)	0 (0%)	2 (4%)	0 (0%)	
Not Hispanic	199 (72%)	103 (76%)	8 (73%)	36 (67%)	52 (69%)	
Race (N = 276)						
African American	60 (22%)	23 (17%)	1 (9%)	15 (28%)	21 (28%)	
Asian	30 (11%)	20 (15%)	0 (0%)	1 (2%)	9 (12%)	
White	122 (44%)	64 (47%)	8 (73%)	23 (43%)	27 (36%)	
Other	64 (23%)	29 (21%)	2 (18%)	15 (28%)	18 (24%)	
Pre-pregnancy Body mass index (kg/m ^2^) (N = 274)						0.130
Mean ± St. Dev.	29.01 ± 7.52	28.39 ± 6.94	27.24 ± 7.55	31.39 ± 8.51	28.70 ± 7.58	
Median; Range	26.98; 17.30 to 51.00	26.60; 17.30 to 50.60	25.39; 20.66 to 46.29	30.00; 19.27 to 49.61	26.93; 20.00 to 51.00	
Maternal age at delivery (years) (N = 276)						0.852
Mean ± St. Dev.	30.41 ± 5.26	30.22 ± 5.35	30.73 ± 4.17	31.07 ± 5.22	30.23 ± 5.31	
Median; Range	31.00; 16.00 to 42.00	30.50; 18.00 to 41.00	31.00; 20.00 to 35.00	31.00; 20.00 to 42.00	31.00; 16.00 to 39.00	
Hypertension (N = 276)						
None	209 (76%)	102 (75%)	10 (91%)	38 (70%)	59 (79%)	
Chronic hypertension	17 (6%)	8 (6%)	1 (9%)	7 (13%)	1 (1%)	
Gestational hypertension	27 (10%)	15 (11%)	0 (0%)	3 (6%)	9 (12%)	
Pre-eclampsia with SF ^3^	10 (4%)	5 (4%)	0 (0%)	1 (2%)	4 (5%)	
Pre-eclampsia without SF ^3^	5 (2%)	3 (2%)	0 (0%)	1 (2%)	1 (1%)	
SIPE ^4^ with SF ^3^	8 (3%)	3 (2%)	0 (0%)	4 (7%)	1 (1%)	
Diabetes (N = 276)						
None	240 (87%)	117 (86%)	10 (91%)	45 (83%)	68 (91%)	
Type 1 Diabetes	1 (0%)	0 (0%)	0 (0%)	1 (2%)	0 (0%)	
Type 2 Diabetes	8 (3%)	3 (2%)	0 (0%)	5 (9%)	0 (0%)	
GDMA1 ^5^	14 (5%)	8 (6%)	0 (0%)	0 (0%)	6 (8%)	
GDMA2 ^6^	13 (5%)	8 (6%)	1 (9%)	3 (6%)	1 (1%)	
Drug use (N = 276)						
Tobacco vaping	3 (1%)	3 (2%)	0 (0%)	0 (0%)	0 (0%)	0.499
Cannabis	26 (9%)	16 (12%)	1 (9%)	3 (6%)	6 (8%)	0.572
Central nervous system stimulants	1 (0%)	0 (0%)	0 (0%)	1 (2%)	0 (0%)	0.236
Central nervous system depressants	2 (1%)	1 (1%)	0 (0%)	1 (2%)	0 (0%)	0.489
Narcotics	4 (1%)	2 (1%)	0 (0%)	2 (4%)	0 (0%)	0.306
History of fetal growth restriction (N = 276)	7 (3%)	3 (2%)	0 (0%)	1 (2%)	3 (4%)	0.836
Maternal history of lupus (N = 276)	1 (0%)	0 (0%)	0 (0%)	1 (2%)	0 (0%)	0.236
History of stillbirth (N = 276)	4 (1%)	3 (2%)	0 (0%)	0 (0%)	1 (1%)	0.843
Gestational age at stillbirth delivery (N = 4)						0.346
Mean ± St. Dev.	19.25 ± 4.35	18.00 ± 4.36			23.00 ± NA	
Median; Range	19.50; 15.00 to 23.00	16.00; 15.00 to 23.00			23.00; 23.00 to 23.00	
History of preterm birth (N = 274)	26 (9%)	11 (8%)	1 (9%)	7 (13%)	7 (9%)	0.673
Type of preterm birth history (N = 28)						0.505
Iatrogenic (induced labor)	7 (25%)	2 (18%)	0 (0%)	4 (44%)	1 (14%)	
Spontaneous	21 (75%)	9 (82%)	1 (100%)	5 (56%)	6 (86%)	
History of pregnancy-induced hypertension (N = 276)	22 (8%)	6 (4%)	0 (0%)	6 (11%)	10 (13%)	0.074

^1^ n (%); ^2^ Kruskal–Wallis rank sum test; Fisher’s exact test ^3^ Severe features ^4^ Superimposed Pre-eclampsia, ^5^ Gestational Diabetes without Medications, ^6^ Gestational Diabetes with Medication.,

**Table 2:** Neonatal demographics and outcomes for COVID vs. non-COVID stratified by trimester of diagnosis.

		Timing of COVID Diagnosis (Weeks)			
	Control [N = 136 ^1^]	<13 w; [N = 11 ^1^]	13–28 w [N = 54 ^1^]	28–42 w, [N = 75 ^1^]	*p*-Value ^2^
Gender (N = 276)					0.092
Female	60 (44%)	5 (45%)	24 (44%)	46 (61%)	
Male	76 (56%)	6 (55%)	30 (56%)	29 (39%)	
Gestation (N = 276)					
Singleton	136 (100%)	11 (100%)	54 (100%)	75 (100%)	
Mode of delivery (N = 276)					0.413
Cesarean Section	49 (36%)	3 (27%)	20 (37%)	18 (24%)	
Vaginal	83 (61%)	8 (73%)	34 (63%)	56 (75%)	
Vaginal birth after cesarean section	4 (3%)	0 (0%)	0 (0%)	1 (1%)	
Gestational age at delivery (N = 276)					0.199
Mean ± Standard deviation	38.24 ± 2.53	38.77 ± 0.95	37.51 ± 3.65	38.61 ± 1.78	
Median; Range	39.00; 24.29 to 41.43	39.14; 37.14 to 40.14	38.71; 20.29 to 40.14	39.00; 31.43 to 41.00	
Gestational age at anatomy scan (N = 269)					0.043
Mean ± Standard deviation	20.70 ± 3.12	19.43 ± 0.90	19.92 ± 2.03	20.63 ± 2.72	
Median; Range	20.00; 16.71 to 36.29	19.29; 18.43 to 21.71	19.29; 17.43 to 28.14	19.93; 17.71 to 35.14	
Estimated fetal weight at anatomy scan (g) (N = 261)					0.236
Mean ± Standard deviation	354.95 ± 147.28	297.90 ± 66.28	351.77 ± 167.58	362.32 ± 144.79	
Median; Range	324.50; 169.00 to 1300.00	285.00; 238.00 to 479.00	300.00; 196.00 to 1044.00	333.50; 177.00 to 886.00	
Anatomy scan percentile (N = 270)					0.674
Mean ± Standard deviation	48.98 ± 25.79	48.80 ± 18.88	54.21 ± 23.42	49.77 ± 24.00	
Median; Range	48.00; 1.00 to 99.00	55.00; 22.00 to 75.00	54.00; 11.00 to 97.00	50.00; 4.00 to 93.00	
Decreased fetal movement (N = 276)	14 (10%)	3 (27%)	9 (17%)	13 (17%)	0.190
Fetal distress (N = 276)	11 (8%)	0 (0%)	4 (7%)	10 (13%)	0.497
Chorioamnionitis (N = 274)	6 (4%)	0 (0%)	1 (2%)	2 (3%)	0.820
Small for gestational age (N = 276)	11 (8%)	0 (0%)	5 (9%)	5 (7%)	0.878
Fetal growth restriction (N = 276)	18 (13%)	0 (0%)	5 (9%)	12 (16%)	0.496
Neonatal Intensive Care Unit (N = 276)	19 (14%)	1 (9%)	8 (15%)	9 (12%)	0.970
Apgar 5 min (N = 276)					0.493
Mean ± Standard deviation	8.69 ± 1.07	8.45 ± 1.21	8.44 ± 1.71	8.68 ± 1.14	
Median; Range	9.00; 1.00 to 9.00	9.00; 5.00 to 9.00	9.00; 0.00 to 9.00	9.00; 0.00 to 9.00	
Ponderal Index (N = 276)					0.417
Mean ± Standard deviation	2.51 ± 0.33	2.59 ± 0.19	2.55 ± 0.27	2.53 ± 0.29	
Median; Range	2.51; 1.62 to 3.30	2.61; 2.30 to 2.91	2.62; 1.73 to 3.37	2.55; 1.99 to 3.23	
Birth Measurements (N = 275)					
Weight %tile (z score)					0.431
Mean ± Standard deviation	−0.13 ± 0.95	0.06 ± 0.70	−0.11 ± 0.88	−0.29 ± 0.75	
Median; Range	−0.27; −1.93 to 2.71	−0.04; −1.00 to 1.51	−0.05; −1.92 to 1.35	−0.24; −2.59 to 1.44	
Head Circumference %tile (z score)					0.010
Mean ± Standard deviation	−0.08 ± 0.96	0.05 ± 0.94	0.00 ± 1.05	−0.49 ± 0.92	
Median; Range	−0.03; −2.36 to 2.91	−0.31; −1.24 to 1.60	−0.08; −2.61 to 2.24	−0.50; −2.36 to 1.60	
Length %tile (Z score)					0.577
Mean ± Standard deviation	0.28 ± 1.12	0.33 ± 0.95	0.11 ± 1.10	0.07 ± 1.00	
Median; Range	0.30; −2.85 to 3.54	0.30; −1.14 to 1.72	0.15; −2.12 to 3.03	0.30; −2.56 to 2.40	

^1^ n (%), ^2^ Kruskal-Wallis rank sum test; Fisher’s exact test.

**Table 3:** Multivariate analysis for the growth outcomes comparing COVID moms by diagnosis time to non-COVID moms adjusting for covariates selected for significant associations with COVID groups at 10% significance level.

	N	Beta ^1^	95% CI ^2^	*p*-Value
Gestational age at anatomy scan	268	−0.02	−0.06, 0.02	0.3
History of Pregnancy-induced Hypertension				0.075
Yes		0.46	−0.05, 0.96	
No				
Groups	268			0.023
COVID-free		—	—	
Diagnosis before 13 weeks		0.15	−0.48, 0.78	>999
Diagnosis between 13 and 28 weeks		0.07	−0.24, 0.39	>999
Diagnosis between 28 and 42 weeks		−0.38	−0.65, −0.10	0.024

^1^ Beta = A standardized beta coefficient compares the strength of the effect of each individual independent variable to the dependent variable ^2^ CI = Confidence Interval.

## Data Availability

Data have been withheld for privacy reasons but can be made available upon reasonable request to the authors.

## Abbreviations

The following abbreviations are used in this manuscript:

EMR	Electronic medical record
REDCap	Research Electronic Data Capture
ZKV	Zika Virus
SARS-CoV-2	Acute Respiratory Syndrome Coronavirus-2
NICU	Neonatal intensive care unit
APO	Adverse pregnancy outcome
PCR	Polymerase chain reaction
IRB	Institutional Review Board
SGA	Small for gestational age
FGR	Fetal growth restriction
PI	Ponderal index
HC	Head circumference
BMI	Body mass index
APGAR	Appearance, Pulse, Grimace, Activity and Respiration
GDMA2	gestational diabetes treated with medication
GDMA1	diet-controlled gestational diabetes

## References

[1] Farrell T., Minisha F., Abu Yaqoub S., Rahim A.A., Omar M., Ahmed H., Lindow S., Abraham M.R., Gassim M., Al-Dewik N. (2023). Impact of timing and severity of COVID-19 infection in pregnancy on intrauterine fetal growth- a registry-based study from Qatar. PLoS ONE.

[2] Narang K., Miller M., Trinidad C., Wick M., Theiler R., Weaver A.L., Mehta R.A., Schenone M. (2023). Impact of asymptomatic and mild COVID-19 infection on fetal growth during pregnancy. Eur. J. Obstet. Gynecol. Reprod. Biol..

[3] Schwartz D.A., Avvad-Portari E., Babal P., Baldewijns M., Blomberg M., Bouachba A., Camacho J., Collardeau-Frachon S., Colson A., Dehaene I. (2022). Placental Tissue Destruction and Insufficiency From COVID-19 Causes Stillbirth and Neonatal Death From Hypoxic-Ischemic Injury. Arch. Pathol. Lab. Med..

[4] Bahrami R., Schwartz D.A., Karimi-Zarchi M., Javaheri A., Dastgheib S.A., Ferdosian F., Noorishadkam M., Mirjalili S.R., Neamatzadeh H. (2021). Meta-analysis of the frequency of intrauterine growth restriction and preterm premature rupture of the membranes in pregnant women with COVID-19. Turk. J. Obstet. Gynecol..

[5] Ishihara N., Matsuo H., Murakoshi H., Laoag-Fernandez J.B., Samoto T., Maruo T. (2002). Increased apoptosis in the syncytiotrophoblast in human term placentas complicated by either preeclampsia or intrauterine growth retardation. Am. J. Obstet. Gynecol..

[6] Schwartz D.A., Dhaliwal A. (2021). Coronavirus Diseases in Pregnant Women, the Placenta, Fetus, and Neonate. Adv. Exp. Med. Biol..

[7] Wei S.Q., Bilodeau-Bertrand M., Liu S., Auger N. (2021). The impact of COVID-19 on pregnancy outcomes: A systematic review and meta-analysis. CMAJ.

[8] Giuliani F., Oros D., Gunier R.B., Deantoni S., Rauch S., Casale R., Nieto R., Bertino E., Rego A., Menis C. (2022). Effects of prenatal exposure to maternal COVID-19 and perinatal care on neonatal outcome: Results from the INTERCOVID Multinational Cohort Study. Am. J. Obstet. Gynecol..

[9] Schwartz D.A. (2022). Stillbirth after COVID-19 in Unvaccinated Mothers Can Result from SARS-CoV-2 Placentitis, Placental Insufficiency, and Hypoxic Ischemic Fetal Demise, Not Direct Fetal Infection: Potential Role of Maternal Vaccination in Pregnancy. Viruses.

[10] Overton E.E., Goffman D., Friedman A.M. (2022). The Epidemiology of COVID-19 in Pregnancy. Clin. Obstet. Gynecol..

[11] Wilkinson M., Johnstone E.D., Simcox L.E., Myers J.E. (2022). The impact of COVID-19 on pregnancy outcomes in a diverse cohort in England. Sci. Rep..

[12] Rad H.S., Rohl J., Stylianou N., Allenby M.C., Bazaz S.R., Warkiani M.E., Guimaraes F.S.F., Clifton V.L., Kulasinghe A. (2021). The Effects of COVID-19 on the Placenta During Pregnancy. Front. Immunol..

[13] Regan A.K., Arah O.A., Fell D.B., Sullivan S.G. (2022). SARS-CoV-2 Infection During Pregnancy and Associated Perinatal Health Outcomes: A National US Cohort Study. J. Infect. Dis..

[14] Pereira L., Petitt M., Fong A., Tsuge M., Tabata T., Fang-Hoover J., Maidji E., Zydek M., Zhou Y., Inoue N. (2014). Intrauterine growth restriction caused by underlying congenital cytomegalovirus infection. J. Infect. Dis..

[15] Walker C.L., Ehinger N., Mason B., Oler E., Little M.E., Ohuma E.O., Papageorghiou A.T., Nayeri U., Curry C., Adams Waldorf K.M. (2020). Ultrasound prediction of Zika virus-associated congenital injury using the profile of fetal growth. PLoS ONE.

[16] Kilby M., Hodgett S. (2000). Perinatal viral infections as a cause of intrauterine growth restriction. Intrauterine Growth Restriction.

[17] Hanshaw J.B., Dudgeon J.A. (1978). Varicella-zoster infections. Major Probl. Clin. Pediatr..

[18] Kuadkitkan A., Wikan N., Sornjai W., Smith D.R. (2020). Zika virus and microcephaly in Southeast Asia: A cause for concern?. J. Infect. Public Health.

[19] Antoniou E., Orovou E., Andronikidi P.E., Orovas C., Rigas N., Palaska E., Sarella A., Iatrakis G., Voyiatzaki C. (2021). Congenital Zika Infection and the Risk of Neurodevelopmental, Neurological, and Urinary Track Disorders in Early Childhood. A Systematic Review. Viruses.

[20] Christian K.M., Song H., Ming G.L. (2019). Pathophysiology and Mechanisms of Zika Virus Infection in the Nervous System. Annu. Rev. Neurosci..

[21] Sanchez Clemente N., Brickley E.B., Paixao E.S., De Almeida M.F., Gazeta R.E., Vedovello D., Rodrigues L.C., Witkin S.S., Passos S.D. (2020). Zika virus infection in pregnancy and adverse fetal outcomes in Sao Paulo State, Brazil: A prospective cohort study. Sci. Rep..

[22] Whitelaw A. (2019). Chapter 3- Posthemorrhagic Hydrocephalus Management Strategies. Neurology.

[23] Karnik M., Beeraka N.M., Uthaiah C.A., Nataraj S.M., Bettadapura A.D.S., Aliev G., Madhunapantula S.V. (2021). A Review on SARS-CoV-2-Induced Neuroinflammation, Neurodevelopmental Complications, and Recent Updates on the Vaccine Development. Mol. Neurobiol..

[24] Wu Y., Xu X., Chen Z., Duan J., Hashimoto K., Yang L., Liu C., Yang C. (2020). Nervous system involvement after infection with COVID-19 and other coronaviruses. Brain Behav. Immun..

[25] Coolen T., Lolli V., Sadeghi N., Rovai A., Trotta N., Taccone F.S., Creteur J., Henrard S., Goffard J.C., Dewitte O. (2020). Early postmortem brain MRI findings in COVID-19 non-survivors. Neurology.

[26] Vimercati A., De Nola R., Dellino M., Vinci L., Ricci I., Malvasi A., Damiani G.R., Gaetani M., Lamanna B., Cicinelli E. (2023). SARS-CoV-2 Infection in the Second Trimester of Pregnancy: A Case Report of Fetal Intraventricular Hemorrhage After Critical COVID-19 Infection and a Brief Review of the Literature. Cureus.

[27] Duppers A.L., Bohnhorst B., Bultmann E., Schulz T., Higgins-Wood L., von Kaisenberg C.S. (2021). Severe fetal brain damage subsequent to acute maternal hypoxemic deterioration in COVID-19. Ultrasound Obs. Gynecol..

[28] Favre G., Mazzetti S., Gengler C., Bertelli C., Schneider J., Laubscher B., Capoccia R., Pakniyat F., Ben Jazia I., Eggel-Hort B. (2021). Decreased Fetal Movements: A Sign of Placental SARS-CoV-2 Infection with Perinatal Brain Injury. Viruses.

[29] Abdelkader M.A., Ramadan W., Gabr A.A., Kamel A., Abdelrahman R.W. (2017). Fetal intracranial hemorrhage: Sonographic criteria and merits of prenatal diagnosis. J. Matern. Fetal Neonatal Med..

[30] Harris P.A., Taylor R., Minor B.L., Elliott V., Fernandez M., O’Neal L., McLeod L., Delacqua G., Delacqua F., Kirby J. (2019). The REDCap consortium: Building an international community of software platform partners. J. Biomed. Inform..

[31] Harris P.A., Taylor R., Thielke R., Payne J., Gonzalez N., Conde J.G. (2009). Research electronic data capture (REDCap)—A metadata-driven methodology and workflow process for providing translational research informatics support. J. Biomed. Inform..

[32] Martins J.G., Biggio J.R., Abuhamad A., Society for Maternal-Fetal Medicine (2020). Society for Maternal-Fetal Medicine Consult Series #52: Diagnosis and management of fetal growth restriction: (Replaces Clinical Guideline Number 3, April 2012). Am. J. Obstet. Gynecol..

[33] Fenton T.R., Nasser R., Eliasziw M., Kim J.H., Bilan D., Sauve R. (2013). Validating the weight gain of preterm infants between the reference growth curve of the fetus and the term infant. BMC Pediatr..

[34] Hwang J.K., Kang H.N., Ahn J.H., Lee H.J., Park H.K., Kim C.R. (2022). Effects of Ponderal Index on Neonatal Mortality and Morbidities in Extremely Premature Infants. J. Korean Med. Sci..

[35] Landmann E., Reiss I., Misselwitz B., Gortner L. (2006). Ponderal index for discrimination between symmetric and asymmetric growth restriction: Percentiles for neonates from 30 weeks to 43 weeks of gestation. J. Matern. Fetal Neonatal Med..

[36] Weaver D.D., Christian J.C. (1980). Familial variation of head size and adjustment for parental head circumference. J. Pediatr..

[37] Mittal S., Federman H.G., Sievert D., Gleeson J.G. (2022). The Neurobiology of Modern Viral Scourges: ZIKV and COVID-19. Neuroscientist.

[38] Steiner M.L., Cunha B.C.R., de Almeida J.F.M., Carrijo G., Dutra L., Suano F., Giovanelli S., Carneiro M., da Silva M.H. (2023). Evaluation of Maternal Fetal Outcomes of Pregnant Women and Mothers with Suspected Infection by SARS-CoV-2 Treated at the Municipal Hospital of Sao Bernardo do Campo (HMU-SBC), Brazil. Matern. Child Health J..

[39] Ockene M.W., Russo S.C., Lee H., Monthe-Dreze C., Stanley T.L., Ma I.L., Toribio M., Shook L.L., Grinspoon S.K., Edlow A.G. (2023). Accelerated Longitudinal Weight Gain Among Infants With In Utero COVID-19 Exposure. J. Clin. Endocrinol. Metab..

